# Communicating risk and the landscape of cancer prevention — an exploratory study that examines perceptions of cancer-related genetic counseling and testing among African Americans and Latinos in the Midwest

**DOI:** 10.1007/s12687-022-00629-5

**Published:** 2023-03-17

**Authors:** Crystal Y. Lumpkins, Rafaela Nelson, Zawadi Twizele, Mariana Ramírez, Kim S. Kimminau, Alisdair Philp, Reem A. Mustafa, Andrew K. Godwin

**Affiliations:** 1 Department of Communication, Huntsman Cancer Institute, University of Utah, Salt Lake City, UT USA; 2 Pediatrics Department, University of Kansas Medical Center, Kansas City, KS USA; 3 University of Kansas Medical Center, Kansas City, KS USA; 4 Department of Population Health, JUNTOS Center for Advancing Latino Health, University of Kansas Medical Center, Kansas City, KS USA; 5 Department of Family and Community Medicine, University of Missouri, Columbia, MO USA; 6 Exact Sciences Laboratories, Madison, WI USA; 7 Department of Internal Medicine, University of Kansas Health System, Kansas City, KS USA; 8 Department of Microbiology, Molecular Genetics and Immunology, University of Kansas Medical Center, Kansas City, KS USA

## Abstract

African American (AA) and Latino populations are impacted disproportionately by cancer incidence and mortality compared to the general US population. Contributing to these rates are multiple inheritable cancers that impact both men and women. Some of these diseases may be detected through genetic counseling and germline DNA testing; however, AA and Latinos are unaware and have limited knowledge and thus significantly underutilize these services and technologies. Research to detect influencing factors to testing uptake has also been slow due to multiple factors. The research team followed a community-based participatory research (CBPR) approach and worked with a Community Advisory Board composed of cancer survivors and co-survivors to design the exploratory study. Six focus groups were held with a pilot sample of African Americans and Latinos who self-reported to be at-risk for cancer (*N* = 53). The study was held over a 2-month period where attitudes, perceptions, and beliefs about cancer risk and preference regarding cancer-related genetic counseling and testing risk communication were explored. Themes that emerged included (1) the lack of knowledge about cancer-related genetic counseling and testing; (2) cancer is feared often; (3) cancer-related genetic testing was perceived as something that could help but was also perceived as unnecessary testing that exposed individuals to medical harm; and (4) benefits to test were perceived as favorable for medical personnel but not for the patient. Implications of the study provide a unique lens to explore how lived experiences among AA and Latinos may inform strategic risk communication about cancer-related genetic counseling and testing and help advance cancer health equity. Participants viewed cancer genetic testing as important cancer risk prevention strategies. Identification of perceptions of cancer risk and cancer-related genetic counseling and testing in collaboration with members of the community is needed to bolster communication efforts among these populations.

## Introduction

In the era of precision medicine, advancements of cancer genetic and genomic technologies have grown exponentially, expanding healthcare (Patch & Middleton 2018) and informing community and public health efforts (Senier et al. 2019). While these technologies are accessible to some, awareness, knowledge of, and integration into clinical care are limited, particularly among racial/ethnic populations where health disparities persist and impede cancer health equity (Alcaraz et al. 2020). African American and Latino populations continue to experience disproportionate cancer incidence compared to most of the US population. The probability of an increased risk of dying from cancer also is higher among these populations (ACS 2019) and further illustrates deficits in cancer prevention and cancer care across the continuum. Contributing to these rates are multiple inheritable cancers that impact both men and women. For example, Latina women show an overall incidence of breast cancer lower than White women. However, breast cancer is often diagnosed at a later stage, and tumors are larger than in White women (DeSantis et al. 2019; Lynce et al. 2016). Among African American women, age-adjusted breast cancer mortality is nearly 40% higher (Jatoi et al. 2022), and there is higher breast cancer incidence before age 40, but incidence is lower between the ages 65 to 84. The likelihood of death from breast cancer however is higher among African American women at every age. African American and Latino men are also disproportionately impacted by cancer, having the highest mortality rate for prostate cancer compared to all other racial/ethnic groups (NCI 2021).

 Approximately 10% of most cancers are attributable to inherited cancer syndromes and significantly elevated where the age at onset is even younger than in the general population (Ricker et al. 2018) (p. 85). Some of these inherited syndromes may be detected through genetic counseling and germline DNA testing prior to a cancer diagnosis; however, there is disproportionate genetic counseling and germline DNA testing among African Americans and Latinos (Canedo et al. 2019; Carroll et al. 2020; Singer et al. 2004) due to several barriers. Mistrust of medical personnel (Corbie-Smith et al. 2002; Singer et al. 2004), limited access to cancer risk assessment (Komenaka et al. 2016) (Ricker et al. 2006), low awareness ( Halbert et al. 2005; Hann et al. 2017; Singer et al. 2004), limited knowledge, and other patient-level, psycho-social factors (Olaya et al. 2009) contribute to low rates of genetic testing in African American and Latino populations. A growing body of research shows how risk perceptions about cancer-related genetic testing among racial/ethnic minorities (Chavez-Yenter et al. 2020; Peterson et al. 2018) may contribute to disproportionate counseling and testing approaches for prevention and early detection of inheritable cancers. Gaps in physician recommendations (McCarthy et al. 2016) could also explain disparities among racial/ethnic minority populations. Identification of individual-level and other structural/environmental barriers serves as a guide for health communication strategy and clinical practice and how to effectively communicate cancer risk among racial/ethnic minority (patient) populations. Available and accessible hereditary disease history information and thus the probability of inheritability of cancer and other risk factors through an inherited cancer syndrome risk assessment are additional pieces of information for informed decision-making between provider and patient (Timmermans 2020). Precision cancer prevention communication that is holistic rather than focused singularly on an oncological lens (Butler et al. 2022) provides an avenue to view multiple factors that may impact cancer prevention health equity.

Cancer risk and prevention in the context of cancer-related genetic testing risk communication is an area of clinical health communication where the inclusion of risk messaging may guide strategy for genetic testing. The present study acknowledges previous research but applies a novel community-engaged approach and to our knowledge is one of the first to inform the development of a risk communication model designed to strengthen genetic counseling and testing efforts and advance cancer health equity information among racial/ethnic minority populations. Drawing from robust literature and identifying psycho-social factors among the pilot sample, we reveal areas that will be critical for a clinical risk communication model to address cancer screening disparities.

## Communicating cancer risk with cancer-related genetic testing among racial/ethnic minority populations

Identification of risk factors for disease is a critical component for calculating and assessing risk (Lautenbach et al. 2013). Those risks are partially determined by modifiable (e.g., lifestyle) and non-modifiable (e.g., genetics) factors. Communicating risk for genetic variations and other health conditions to the public presents multiple challenges including limited awareness and knowledge about basic genetic and genomic concepts among patients and clinicians; minimal understanding of the influence of family health history among those at risk; misinformation or distortion of information through mass media and direct advertising; and overall gaps in communication or cancer risk on multiple levels (Parrott et al. 2015).

Risk communication at an individual level enables those at a predicted increased risk of developing cancer to make informed decisions. Understanding risk perception about cancer and how these perceptions influence genetic counseling and testing among diverse populations could help guide effective clinical and public health communication strategies to address testing disparities. Judgment of risk acceptability also impacts how individuals make decisions about their health and actively seek care. The level of risk and how they evaluate and interpret the risk are influenced by multiple factors (i.e., fairness, benefits, alternatives, control, voluntariness) and will determine acceptance (Covelo 1991). Tailored approaches which include absolute risk or risk that is relevant compared to non-tailored approaches are more effective (Lautenbach et al. 2013; Kreuter et al. 2003). These approaches have the potential to increase salience of information and to positively impact attitudes, perceptions, and beliefs about the value of cancer-related genetic counseling and testing. Furthermore, culturally inclusive approaches maximize opportunities for equitable knowledge gathering, health information sharing, and dissemination.

Identification of attitudes, perceptions, and beliefs among racial/ethnic populations is critical for culturally appropriate cancer communication (Kinney et al. 2010; McQueen et al. 2011). Lack of culturally appropriate cancer-related genetic testing information (Peterson et al. 2018) and counseling risk communication strategies among minority and underserved populations (Jones et al. 2016; McCarthy et al. 2016; Smith et al. 2016) are contributors to disparities among these populations (Chavez-Yenter et al. 2020; Pagán et al. 2009). Furthermore, barriers such as genetic counselor bias, lack of diversity within the profession (Price et al. 2020), mistrust for medical personnel, access to health care, and education (Halbert et al. 2005) contribute to these disparities. Observational research shows gaps exist among racial/ethnic populations and attitudes, perceptions, and beliefs that hinder testing (Peterson et al. 2018), but more can be gained through engagement of and with community members from these populations; contribution to equitable cancer education and clinical communication models is also plausible. Mounting evidence demonstrates added value aspects of conducting research in partnership with non-research–trained stakeholders (Domecq et al. 2014), and much of the literature points to the importance of establishing trustworthy, bi-directional communications to achieve outcomes (Roche et al. 2020; Harrison et al. 2019).

The research team followed a community-based participatory approach (CBPR) which rests on a continuous establishing a trusting relationship between the researcher and patient partner(s). In this study, CBPR was the platform chosen to explore attitudes, perceptions, and beliefs toward cancer and cancer-related genetic counseling and testing among a pilot sample of African Americans and Latinos. Additionally, participants shared their views on cancer-related genetic counseling and testing (i.e., genetic susceptibility testing) risk communication as a cancer risk prevention strategy.

## Methods

### Study design

Data were collected through a CBPR process that included forming a specific study community advisory board (CAB) from other existing African American and Latino CABs. The CAB guided the research focus and informed the research design and consisted of two African American cancer survivors and one Latina cancer co-survivor (an individual who lends support beginning at diagnosis through treatment). The research team included a genetic counselor, a precision medicine scientist, two social scientists, the director of a Latino center for health, and one physician scientist. Focus groups were conducted to collect data on participants’ experiences with cancer and cancer-related genetic counseling and testing and their perceptions about cancer-related genetic counseling and testing risk communication. The Theory of Planned Behavior (Ajzen 1991) guided focus group discussions where the constructs (attitude, subjective norms, perceived behavioral control) were used to explore participants’ attitudes, perceptions, and beliefs about cancer and cancer-related genetic counseling and testing. The team used Covelo’s risk perception model (Covelo 1991) to guide the discussion about communicating risk for cancer and genetic susceptibility testing. These constructs guided the analysis and exploration of how participants’ lived experiences shaped their perceptions about cancer risk based on inherited factors and their likelihood to explore genetic counseling and testing. The team also used a previous pilot study’s findings as a guide for the present study (Lumpkins et al. 2020). The University of Kansas Medical Center Institutional Review Board (IRB #00142461) approved the study.

### Study population

The study team recruited focus group participants in 2019 using existing community networks. Individuals eligible to participate in the study self-identified as either African American and/or Latino, were able to communicate in English, were at least 18 years old, self-identified as a cancer survivor or cancer co-survivor, identified as being either high or moderate risk for cancer, or had a family history of cancer.

### Engagement with CAB

The CAB met monthly with the research team between October 2018 and March of 2019 to discuss the research design. CAB members reviewed a moderator’s guide from a previous study conducted with African American faith populations (Lumpkins et al. 2020) and developed a guide for the study with African American and Latino populations. Members also agreed and finalized the study’s focus group recruitment strategy, focus group procedures, and survey. The semi-structured guide included the following four domains of interest (see Table 1): (1) experiences with cancer and genetic testing for cancer risk; (2) Cancer-related genetic counseling and testing; (3) Cancer-related genetic counseling and testing risk communication; and (4) Barriers to screening.

**Table 1:** Focus group moderator’s guide

Topic	Questions
1. Experiences with cancer and genetic testing for cancer risk	When you hear the word cancer, what comes to mind? What feelings or reactions do you have when doctors and nurses talk about *just checking* for cancer? What do you think can be done if a genetic test informs your doctor that you have an increased risk for cancer? What are your thoughts about finding out about genetic risk in your family?
2. Cancer-related genetic counseling and testing	What comes to mind when you think about genetic counseling? What comes to mind when you think about genetic testing?
3. Cancer-related genetic counseling and testing risk communication	When you think of genetic counseling and testing communication, what are your thoughts about benefits? Risks? Barriers? What are your thoughts about genetic counseling and testing communication that is shared within your family; between you and your medical provider; shared in society?
4. Barriers to screening	How does cost figure into the decision of whether to get genetic counseling or genetic testing? How comfortable would you be talking to family members about genetic counseling or testing? What about talking to a medical professional? How comfortable would you be if your genetic test results were in your medical records?

### Recruitment

A purposive sampling technique was used to recruit from within African American and Latino networks in the Kansas City metropolitan area with the support of the CAB; JUNTOS Center for Advancing Latino Health (JUNTOS), a community-academic partnership to improve Latino health; and Faith Works Connecting for a Health Community (FWCFHC), a consortium created to address cancer disparities among African American faith communities. This process included posting recruitment flyers on the JUNTOS social media page and community-based locations and flyers handed out to FWCFHC consortium members during meetings and announcements given by church pastors and health ministry leaders during worship service and other church activities. Recruitment occurred from January to March 2019. Three research assistants reflecting the study population called or emailed individuals to determine eligibility, answered questions, and enrolled participants in specific focus group sessions (*N* = 6).

### Focus group procedures

The study team held focus groups between March and May of 2019 at the University of Kansas Medical Center campus with easily accessible parking and public transportation access for participants. Participants were given a written, informed consent document prior to the start of the focus group. A study staff member also engaged in conversation and answered questions. Prior to the start of the focus group, participants completed a survey about cancer screening, genetic counseling, and genetic testing intention and completion. Study staff that included a moderator, co-moderator, and note-taker assigned participants in a circular seating arrangement. Three African American and 3 Latino focus groups were held between 70 and 90 min and included 4 to 13 participants in each group. Participants received a $25 gift card as an honorarium. The research team recorded all but one of the focus groups and subsequently transcribed recorded discussions for data analysis. One African American focus group discussion recording was missing from the analysis because of audio recording failure; however, the team included focus group notes in the analyses. The principal investigator, co-investigator from JUNTOS, translational scientist, and physician scientist reviewed each transcript for analysis.

### Analysis

Three social science researchers and one physician scientist followed an open coding and constant comparison method (Denzin and Lincoln 2011) to identify themes. Coders met between October 2019 and May 2020 to analyze the collected data. The coders first individually analyzed transcripts and subsequently came together to discuss and form a consensus for overarching categories and phrases, words, and a preliminary identification of codes and themes. A score sheet was subsequently created to quantify how phrases appeared and fit within these categories during each focus group discussion. After coders completed this step, the research assistant compiled the data, and the coders subsequently met as a team one final time to discuss the categories and emerging themes across all focus groups. From this analysis, coders identified five themes.

## Results

Focus group discussions were guided by a semi-structured interview guide (Table 1) and mirrored survey results that illuminated social determinants of health that impede cancer health equity (Alcaraz et al. 2020). Key themes that emerged about cancer-related genetic counseling and testing and communication of testing technology among racial/ethnic minority communities (Table 2) also yielded identifiable factors (individual and structural) that hinder equitable precision cancer prevention (Butler et al. 2022) among the study sample (Table 3). Participants were primarily female (92%), had at least a high school education (76%), and had not seen a genetic counselor (87%) even when self-reporting as high or at moderate risk for cancer (Table 4). Coders identified five overarching themes through their analysis. First, participants had limited knowledge about cancer-related genetic counseling and testing. They also shared a fear of cancer, believing that cancer is (often) fatal. Another theme that emerged from focus group discussions included the perception of risk associated with cancer-related counseling and testing; this perception was a barrier to participating in cancer-related genetic counseling and testing. Participants also saw the benefit to participate in counseling and testing as a benefit for others. Finally, participants believed it was important to culturally tailor this type of information.

**Table 2:** Sample quotes from focus group

Categories	Participant quotes	
1. Knowledge gap about cancer-related genetic counseling and testing	AA	“I wonder why it was a question at all if genetic testing should be available because if there is a way to just be knowledgeable about what is going on with your body, anybody, I would think that would just be automatic, instead of just being offered now.”
	L	“I’m 43 and I have never been offered a genetic test and it’s, you know, I have been sitting here thinking about I have been going to the same person or the same clinic for 10 years and never once have they said maybe we should do this or maybe we should do that.”
2. Cancer is feared and (often) fatal	AA	“I (also) think of death, it’s like there is nothing to help. The result is going to be death.” “…if they were given that death sentence that is how I take it, at least give them some hope.”
	L	“Limited time. You have(an) expiration date.” “It is unexpected and whatever happens it takes life really fast.”
3. Perceived risk of cancer-related genetic testing	AA	“I feel like I am a test bunny or a lab rat.” “Who is getting the data and that results (…) how are you insuring that is not going to be used for some side business that they want to sneak with?”
	L	“… they think that if you do the genetic testing, that is going to diagnose to whether or not you are going to have a cancer.” “I don’t want to be a guinea pig for you.”
4. Limited benefits to participate in cancer-related genetic counseling and testing	AA	“It doesn’t necessarily cure anything it just lets you know what runs in your lineage.” “What would that do for me? It would do nothing for me.”
	L	“It’s the fear, because of the misconception of what that is going to be doing (…) they think that if you do the genetic testing, that is going to diagnose (…)” “(…) it doesn’t address the benevolence.” “They even tell you that ‘Don’t worry we are going to pay you forever…so what?’ I am going to be your guinea pig forever. No way, Jose.”
5. Cancer-related genetic testing communication must be culturally tailored	AA	“They just need somebody to explain it to them (…) in a way that they can understand because that is just something else.” “Start networking then we can exchange information and support each other.” “Communicating in plain simple clear language, the same thing that everybody else couldn’t get.” “(…) in the black culture, you wouldn’t even know why, that would be grown folk’s business.” “Are you explaining it not just in medical terms, but in the vernacular that they can understand?”
	L	“Education, information is power.” “… explain what is going to happen and why is this done and what are the benefits not for yourself but for the community. I think that more people would do it.” “Come and talk to my level and tell me the right thing.” “(…) explain things on the radio, something on the paper… You have to go out and spread the word and explain as much and as easy as you can.”

*AA* African American, *L* Latinos

**Table 3:** Socio-demographic characteristics of participants (*N* = 53)

African American and Latino groups		Age Mean (*x̅*)			Both groups combined (*N* = 53)	
Age	African American	(*n* = 24)	49	AA and Latinos combined	45.16	
	Latino	(*n* = 29)	42			
Gender	African American	Female	92%	AA and Latinos combined	Female	79%
		Male	8%			
	Latino	Female	59%		Male	21%
		Male	41%			
Education	African American	High school/GED	71%	AA and Latinos combined	High School/GED	76%
	Latino	High school/GED	76%			
Health coverage	African American	Yes	29%	AA and Latinos combined	Yes	62%
		No	71%		No	38%
	Latino	Yes	90%			
		No	10%			

*AA* African American; *L* Latino

**Table 4:** Genetic testing survey

Cancer-related genetic testing information	Responses			
Ever talked with a doctor or health care provider about getting a genetic test?	African American	No	87.5%	African American and Latino combined
		Do not know/not sure	4.2%	
	Latino	No	86.2%	
		Do not know/not sure	3.4%	
Believe high at risk for cancer	African American	Yes	33.3%	African American and Latino combined
		Do not know/not sure	29.2%	
	Latino	Yes	31.0%	
		Do not know/not sure	20.7%	
A family member had genetic testing	African American	Yes	65%	African American and Latino combined
		Do not know/not sure	35%	
	Latino	Yes	10.3%	
		Do not know/not sure	31.0%	

African American (*n* = 24); Latino (*n* = 29)

### Themes

#### Knowledge gap about cancer-related genetic counseling and testing

There was an overall knowledge gap for cancer-related genetic counseling and testing among both African American and Latino focus group members. Participants drew from their experiences as cancer survivors, cancer co- survivors, and those who self-identified as at risk for cancer.

“I think that it is good reason for genetic testing because I hear women say she had breast cancer and then her momma had it and her sister had it, I think when it’s hitting family like that I think that genetic testing would be good because they would benefit from it because they would be aware before they even end up with the symptoms…” (African American participant, 4–16-19).

“I wonder why it was a question at all if genetic testing should be available because if there is a way to just be knowledgeable about what is going on with your body, anybody, I would think that would just be automatic, instead of just being offered now.” (African American participant, 3–30-19).

“I’m 43 and I have never been offered a genetic test and it’s, you know, I have been sitting here thinking about I have been going to the same person or the same clinic for 10 years and never once have they said maybe we should do this or maybe we should do that.” (Latino participant, 4–17-19).

#### Cancer is feared and (often) fatal

Participants responded that their experiences with cancer were associated with finality of life, i.e., “it (cancer) was a death sentence.” This observation is consistent with an existing body of literature that reports perceived cancer fatalism reduces an individual’s desire to pursue cancer prevention screening (Powe and Finnie 2003). Among racial/ethnic populations, fear also presents and perpetuates barriers to cancer treatment, therapies, and throughout the cancer continuum (Dettenborn et al. 2004).

“Cancer, it scares me; yes, it just scares me. I lost my sister to cancer and a close friend; my feeling is just they are automatically dead.” (African American participant, 4–16-2019).

“Limited time. You have an expiration date.” (Latino participant, 4–17-2019).

“Fear, a lot of times when people are diagnosed with cancer, fear creates a lot of things in their mind, in my opinion, you can’t do anything about it.” (African American participant, 3–30-19).

“When you hear about cancer, we are scared and now I remember years ago in Mexico, everybody went talking about cancer and thinking we will die, maybe one month, two month and it’s very sad.” (Latino participant, 5–9-19).

A sub-theme of spirituality informed perceptions about cancer and testing also emerged among African American focus groups.

“What he (the physician) said he would do; I don’t agree with it. He knows that I don’t agree with it. So, I already know I am going to talk to my spiritual healers, my people and see what they think I should do.” (African American participant, 4–16-2019).

“You have to believe that God loves you. You have to believe that there is somebody bigger than you and that allows you to wake up in the morning. If you don’t believe that, it’s easy to say a lot…if you don’t walk in that faith.” (African American participant, 4–16-2019).

#### Perceived risk of cancer and cancer-related genetic counseling and testing

Participants saw the discovery of cancer risk through medical practice and research as valuable for cancer prevention. They also saw it as an avenue to surreptitiously extrapolate data and information from vulnerable individuals and the potential to expose them to harmful practices that lead to cancer. Participants discussed how medical practice and research agendas may expose them to harmful practices that lead to cancer.

“My mom died in Mexico; it was 1996. The doctor said they would like to give a test just for (us) girls; we said no, we don’t want to be a guinea pig. I’m sorry, we didn’t know what kind of cancer my mom had but I don’t want to be a guinea pig for you, sorry no. And the four of us were like I won’t do it.” (Latino participant, 5–17-19).

“Don’t bull “stuff” things with me when it comes to medical. They can say it all they want to (genetic information) but I don’t know…so when you are bringing up other stuff, you are letting them know you are trying to dodge and weave the focus point of what we are doing.” (African American participant, 4–16-19).

“It’s the fear, because the misconception of what the test is going to be doing, you know they think that if you do the genetic testing, that it’s going to diagnose to whether or not you are going to have a cancer and that’s not it. You know what I mean, it’s the lack of education is going to be doing, what kind of information it is going to provide. So, I think it’s the misconception is what it is that’s what prevents people from doing it.” (African American participant, 4–16-19).

The overall sentiment was that the “test” had more to offer the scientific and medical establishments rather than provide direct value to the individual. These two racial/ethnic groups differed slightly where Latino participants expressed concern for being a target for research experiments.

“There was a huge resistance in my family to get the test done and it happens here, and it happens in Spain where the doctors are pushing it like a lot and to me it was painful to see my family and I include myself on it because I didn’t do it either, but you see the ones that were most to have it because this was more on my mother’s side not on my father’s side.” (Latino participant, 5-17-2019).

African American participants were especially wary of doctors and mistrustful of the process. They were disappointed about limited awareness and knowledge of this type of information and testing.

“I feel like the power of the many is in the hands of the few. And they don’t look like you, they are not at your level, they are the six people sitting at the head office who, hey I am friends with this head of the pharmaceutical company, we know the FDA thing.” (African American participant, 4–16-19).

#### Limited benefits to participate in cancer-related genetic counseling and testing

Participants discussed the benefits to cancer-related genetic counseling and testing and the direct tie to the benefits of others (medical personnel/researchers), however not for the receiver/patient. The team found that even if the genetic counseling and constitutional DNA testing was offered at no cost, the long-term benefits from the information provided was seen as only leading to additional complications and burdens for the participant. Individuals within the sample felt their limited awareness about the test minimized the rationale to participate in testing and negatively impacted how they felt *others* interpreted and could use the test for cancer prevention and risk. Participants were also concerned how this information would help them after the study along with the burden of additional costs associated with positive results from counseling and genetic testing (e.g., cost for further diagnostic testing, surgery, treatment).

“Open, being super honest about it. If you are going to explain what is going to happen and why it is done and what are the benefits not for yourself but for the community, like you are going to get like twenty bucks for this. We are not silly, we are not dumb, so it’s not about the money, like why is this important?” (Latino participant – May 9, 2019).

“I just want to make sure you are not going to take my information and sell it to someone else. I want to make sure when I give you my genetic, my genetic makeup, this is what my DNA looks like you are not going to, oh let’s just pull that one black lady that now everybody has got genes off of her body.” (African American participant – 3–30-2019).

A sub-theme among Latino groups was also the fear of their immigration status.

“They wanted to scare us and do it too because I am an immigrant and we (are) scared to go see any doctor and they say no. I am an immigrant, and I am scared but that is one of the things that happened to us, especially here in the hospital.” (Latino Focus Participant 5–9-19).

“They explain it that they are not going to share their names but if not, they are just like, ok why do you want to know so much about me? Are you going to implicate me?” (Latino participant – 5–9-19).

#### Cancer-related genetic counseling and testing communication must be culturally tailored

Culturally centered communication was a cross-cutting theme across all focus group discussions. Latino group discussions included specific suggestions for how messages should be created, should be inclusive of culture, and disseminated. Participants believed risk messages about cancer-related genetic counseling and testing would have a greater impact if they were central to everyday life and not cumbersome or an unnecessary inconvenience.

“It’s not just speaking Spanish, than to understand the culture. Others have to understand our culture, it’s not the same to speak Spanish, than to understand there’s like Columbians are different than Mexicans, are different than Venezuelans, we are not the same, we are really, really different.” (Latino participant, 5–9-19).

“I think they should have someone that is a real good speaker that can pinpoint everything and explain it to them because a lot of people are like this doctor stuff can be real.” (African American participant, 4–16-19).

Another participant from a Latino focus group mentioned the importance of a medical provider as the purveyor of this type of information.

“Come talk to my level, and tell me the right thing, don’t tell me with big words because I don’t understand them, and I think this is why people don’t get tested. I don’t want to be a guinea pig for nobody; I don’t care how much you pay me. So, educate first, educate people, the educated people are the ones that we don’t understand, tell me in my language what you are doing with my body or my blood, that’s my body, that’s my right.” (Latino participant – 5–9-19).

## Discussion

This study aimed to explore an under-studied area regarding cancer-related genetic counseling and testing risk communication through a novel approach by focusing on minority and underserved populations with an undetermined risk for any type of cancer. The primary aim was to engage with members from the CAB to design a study to explore attitudes, perceptions, and beliefs toward cancer-related genetic counseling and testing risk communication among Latino and African American patient populations. Our findings highlight the need for increased awareness, knowledge, clear messaging, and transparency from the medical establishment that addresses cancer beliefs and prepares the participant on what cancer-related genetic counseling and testing may reveal.

This pilot sample was primarily composed of educated women who had never had (or been offered) genetic counseling or a germline DNA genetic test. We found that financial-related fears, i.e., loss in wages following a positive test or indicated increased risk associated with an inherited cancer syndrome, could negatively impact interest and counseling and testing completion. Although not an overarching theme, there were individuals in two focus groups who disclosed medical education backgrounds and knowledge during the focus group session and voiced concern about genetic testing and why there was hesitancy to test. Additional research should continue to fully understand how racial/ethnic minority patient populations understand, perceive, and view cancer risk in the context of cancer-related genetic counseling and testing, who is best positioned to provide this service and what barriers may be encountered.

Risk perceptions about cancer and perceptions about medical and research personnel who do or do not inform, communicate, and educate patients about cancer-related genetic counseling and testing have broad implications. Results from a brief survey administered prior to focus groups showed that most participants responded that their medical provider, if they had one, did not inform them of cancer-related genetic counseling/and or testing even where current or past family history was concerning. Some participants also expressed in focus group discussions that they felt the lack of provider communication about this type of testing only reinforced mistrust of medical personnel and underscored documented barriers that perpetuate poor genetic testing use among racial-ethnic minority populations (Underhill et al. 2016). Mistrust may create barriers to delivery of health care and health care services and indifference to perceived susceptibility, perceived benefits, and medical providers and research (Corbie-Smith et al. 2002). In addition, some African Americans and Latinos showed less interest in participating in genetic testing because of their faith and belief in God and their fear of stigmatization and discrimination (Suther and Kiros 2009). Mai and colleagues (Mai et al. 2014) found that in a large sample of non-Hispanic whites, 25 years and older (49.9%) compared to African Americans (32.9%) and Hispanics (20.6%) reported some knowledge of genetic testing to assess their risk of developing cancer (Pagán et al. 2009; Wideroff et al. 2003), but knowledge disparities still exist (Hann et al. 2017; Weise et al. 2021). Mistrust of the health care system providers and medical research in addition to psycho-social barriers, unawareness, lack of knowledge, access to health information (Pagán et al. 2009), and health literacy (Pagán et al. 2009; Singer et al. 2004; Peterson et al. 2018) also are documented contributors to poor cancer-related genetic testing (Pagán et al. 2009; Suther and Kiros 2009) and are consistent with our findings. Addressing these barriers could inform efficacious and evidence-based tailored health communication that resonates with individuals and incorporates individual risk perception from underserved communities.

The perception about disease (cancer) and how individuals may perceive cancer prevention and treatment and evaluate risk information for counseling and testing among racial/ethnic populations was central to our investigation. Gauging how individuals perceive cancer risk and how this type of risk is presented strategically in genetic counseling or testing information may address barriers. Important work in this space (White et al. 2012; Masters and Hooker 2013; Martina et al. 2022) highlights cultural contextualization as critical to addressing cancer screening, outreach, and intervention, but less attention has illuminated these cultural issues that impinge on barriers and facilitators related to genetic counseling or testing.

### Risk communication solutions and strategies

Identification of individual-level barriers including distrust of medical personnel and the fear of cancer among racial/ethnic minorities in the context of cancer-related genetic testing bolsters message relevance. Inequitable communication *about* cancer risk and lack of culturally relevant approaches *for* promulgating genetic counseling and testing among these populations (Jones et al. 2016; McCarthy et al. 2016; Smith et al. 2016) (e.g., genetic counselor bias, mistrust for medical personnel, access to health care, education) (Halbert et al. 2005) contribute to utilization disparities among these populations. Here, the disconnect between health providers and patients and the limited number of trained and licensed genetic counselors are other areas to be explored. 

This sample saw the benefit of genetic testing but also had reservations for reasons why medical personnel would want to offer testing to racial/ethnic minorities. Cancer-related genetic testing risk communication that is culturally inclusive (Peterson et al. 2018; Viswanath et al. 2012) and is tailored to address cultural beliefs among these populations has the potential to offer personalized medicine opportunities to combat cancer health disparities. Community engagement and work through community health workers also hold promise as a strategy to disseminate this type of information. Community health workers or community lay advisors are traditionally trusted within the communities that they serve and are trained to discuss multiple health issues with community members (Almeida et al. 2021; Community Health Workers: Part Of The Solution 2010; Gwede et al. 2013; San Miguel-Majors et al. 2020; Sharpe et al. 2018; Vadaparampil et al. 2021).

Participants also detailed specific message tailoring tactics to improve medical provider (e.g., counselor, doctor) interactions and suggested multiple ways to inform individuals about genetic counseling and testing opportunities. An index used in public health communication helped guide our understanding of how participants could perceive and evaluate a public health risk (Covelo 1991) in terms of cancer and how cancer-related counseling and testing would mitigate that risk. Latino focus group participants provided detailed suggestions for strategies that included family communication, increasing awareness through targeted communication efforts at school and community events, and adopting appropriate language (e.g., speaking and writing in Spanish) for testing information.

Lautenbach and colleagues outline multiple evidence-based strategies to communicate genetic risk information for common medical disorders (Lautenbach et al. 2013). Presenting information in a variety of formats, avoiding framing bias (positive/gain vs. negative/loss), usage of graphics, accounting for cultural beliefs, past experiences and perceived risk, and engaging patients through risk assessment tools are all documented ways to communicate genetic risk information (Lautenbach et al. 2013). More recently, an emphasis is on addressing the influx of genetic information and broad dissemination through multiple channels that include the mass media and advertising (Patch and Middleton 2018). The changing landscape and evolving nature of testing calls for strategies that are responsive to multiple populations.

### Ethical considerations and limitations

There were ethical considerations as well as limitations to the study. Ethical considerations originating from the target communities arose as perceptions about this type of research and testing (genetic) were beneficial to researchers and clinicians, and not patients. African Americans and female participants in the present study voiced outrage that there were cancer prevention tools available but had no awareness of counseling or testing before or during their cancer experience. While these perceptions are like those reported in the literature (Adams et al. 2015; Jones et al. 2016), there are unique points to this study. A small percentage of participants were from a single family and members of community organizations; some family members may have felt pressured to conform to more dominant individuals of the family. Moderators were diligent in including all participants throughout the focus group sessions as some participants were hesitant to speak during parts of the discussion. One African American focus group session (April 6, 2019) was not recorded, and subsequently, data was not transcribed for final analysis. However, the moderator and research assistant were able to use field notes for the coding process. Data from this and most focus group discussions included an overwhelming theme of disenfranchisement, mistrust and distrust of medical doctors, and the lack of transparency among researchers and medical professionals. In addition, the sample was mostly female, especially among the African American focus group discussions; had health insurance; and had completed high school or a GED. A few of these factors may have led to the consensus of mistrust and distrust and how women may perceive cancer throughout the continuum (Molina et al. 2015; Mouslim et al. 2020). Additionally, those who are underinsured or uninsured and have less education may have different perspectives and experiences not represented in the study sample. These additional perspectives may be more representative in Latino populations who are under-represented and uninsured in the nation. Finally, among the Latino-focus groups, the discussions were conducted exclusively in English because of budget limitations to translate research materials and transcripts. Recruitment of English-speaking and bilingual Latino participants may have contributed to a sample of higher educated, higher-income, insured Latino participants. Therefore, results do not reflect additional barriers that many Spanish-speaking Latinos may face (e.g., language, lower access to care, lower literacy level).

To address gaps in knowledge about this type of genetic susceptibility testing and counseling, the research staff and CAB offered free informational sessions following focus group sessions. The team wanted to provide participants and family members of focus groups an opportunity to ask additional questions and access to complementary genetic counseling and testing with genetic counselors on the research team. The study team also disseminated results of findings within established community-based networks to identify ways to improve awareness, knowledge, and information dissemination to decrease genetic testing disparities within these populations.

## Conclusion

To our knowledge, this is one of the first studies to apply a community-engaged approach to inform the development of a risk communication model designed to strengthen genetic counseling and testing efforts among racial/ethnic minority populations. This study added to the growing body of precision cancer health equity literature that focuses on awareness and knowledge of cancer-related genetic counseling and testing among racial/ethnic minorities. Improved access to germline DNA testing may detect inheritable cancer syndromes; however, these populations have limited access to testing due to multiple barriers including physician recommendation, limited awareness and knowledge, and perceptions hindering equity in precision cancer prevention. Results from this study, which assembled a cohort of African Americans and Latinos from a city in the Midwest, are representative of gaps in cancer communication nationally and point to a need for additional targeted communication for these racial/ethnic minority populations. Our findings demonstrate that there are essential factors that must be included as part of cancer risk communication. Communicating effectively about cancer risks requires interdisciplinary best practice and input from engagement with a diversity of cultures and patients. Furthermore, this study demonstrates that communications about cancer risk require greater exploration by individuals focused on clinical benefits and utility for racial/ethnic minority populations. The results from our study support and advance previous literature that demonstrates racial/ethnic minority groups are often disproportionately impacted by limited access and/or awareness of cancer-related counseling and testing (Smith, Fullerton, Dookeran, Hampel, Tin, Maruthur, Schisler, et al., 2016). Additional research that engages the community will continue to inform clinical health communication strategies that are relevant and appeal to underserved and diverse populations and increase the reach of public health communication and emerging precision public health efforts. In an effort to help bridge study participants and their families to counseling and testing, two 90-min informational sessions were held at a centrally located Latino community center and cancer support center near predominately African American neighborhoods in mid-town Kansas City in July and August of 2019. Co-authors were also part of creating the Center for Genetic Services and Health Equity the following year in 2021 that facilitated access to racial/ethnic minorities and under-insured individuals in the urban core of the Kansas City Metropolitan area.

Exploring existing attitudes, perceptions, and beliefs toward cancer-related genetic counseling and testing risk communication among minority and underserved patient populations was the focus of this exploratory study. Based on our previous study of African American faith populations (Lumpkins et al. 2020) and our current community-engagement work with Latinos and African American populations who did not self-identify as from faith communities, we may better define beliefs (and access) and identify specific attitudes and risk perceptions toward cancer and cancer-related genetic counseling and testing among racial/ethnic populations. We also have some understanding for how this type of risk communication may be tailored and disseminated to individuals, groups, and on societal levels. This study will serve as a building block to bolster risk communication strategies that address cancer health inequities, communication inequities, and improvement in genetic testing.

Lessons learned here are both relevant for public health communication research and programmatic health promotion. The community networks were not only important for recruitment of individuals into the study but also for dissemination of information following data collection. These networks may also serve as communication infrastructure that will help build trusted networks of information and knowledge among these populations. Programmatically, public health communication programs that are inclusive of community members where their opinions and input are integrated have implications for bolstering evidence-based practice. Lessons may also be gained from current public health testing issues. Improved messaging regarding the importance and availability of SARS-CoV-2 testing among racial/ethnic populations may also be applied here as we see novel testing technologies unveiled and rapidly disseminated to reach the masses (Khoury and Holt 2021). However, existing beliefs, distrust, and false information have kept many racial/ethnic populations test averse and vaccine hesitant. Culturally inclusive strategies and counseling and testing risk communication that is inclusive of and sensitive to cultural factors have the potential to offer personalized medicine opportunities to combat barriers and cancer health disparities.

## Data Availability

The authors confirm that the data supporting the findings of this study are available within the article [and/or] its supplementary materials. Will Share upon Request.
